# Medicine Men of the World

**Published:** 1887-10-08

**Authors:** 


### Medicine Men of the World.

By the Man in the Crowd.

### I.—INTRODUCTORY.

The medicine-man is all but ubiquitous. Wherever there are human beings, there, we may be assured, will be some sort of a sphere for those who can cure, or will plausibly pretend to cure toothaches and spasms, fevers and agues, epilepsies and bilious attack, colds and neuralgias. Everywhere there are doctors and doctor's bills—savages, by the way, always pay up promptly : it is a mark of high civilisation to have an unpaid doctor's bill about the house—and though over a large portion of the world the practice of medicine and surgery is not quite so scientific as could be wished, nevertheless we have it on the testimony of missionaries and other travellers that if there are any spots on the face of the earth where even medical quacks are not to be found, there are usually to be found substitutes a good deal worse.

Among the savages of the South Sea Islands, for instance, there appear to have been no doctors at all when they were first visited by Europeans. If a man fell sick, the only resource for him was to sacrifice some healthy person's life, and so appropriate his vigour, and the belief of the Islanders was that the nearer the relationship with the individual sacrificed, the more certain the benefit. Among the stories told by the early missionaries here was one of an old chief, who when disease overtook him sent for his younger son, whom he had resolved to have slaughtered as a means of prolonging his own life. The youth, when brought into his father's presence, was seized, and then for the first time began to understand what was designed for him. He struggled desperately, and beat off his assailants. Others, however, came to their aid, and among them the lad's own sister helped to slay him before his father's eyes. Among the Patagonians it is said to be—or it was in the early days of missions—a common thing for a son to be sacrificed to recruit the health of a sick father. Terrible savagery this! We never do such things. The utmost we ever do in this way is to sacrifice a daughter when the social fortunes of the family are in a sick and debilitated condition. But suppose among ourselves custom permitted a son pecuniarily sick and exhausted to resuscitate his financial health by sacrificing a father—after a civilised fashion, of course—who would bet his money that within any given period as many savages would not be found resorting to this sort of doctoring in England as there ever were in Patagonia or the South Sea Islands?

This idea of the transmigration of vital energy from the slain to the slayer is not an unfamiliar one among savage people. It is a common belief that the strength and courage of the warrior who falls fighting become the heritage of the antagonist who slays him ; and it is perhaps even more common for a savage who falls sick to assume that his ailment is due to the bewitchery of somebody who must be slain for the mischief he has done. Not infrequently, too, illness is attributed to the anger of some god, who must be appeased by human sacrifice. But the peculiarity in the custom of the South Sea Islanders and of the Patagonians appears to have been that the slaughter of a son was resorted to merely as an act of appropriation, and as a means of gaining health and strength, which in all other uncivilised countries, nearly, have usually been sought at the hands of medicine-men.

It is interesting to note in passing that it was among the missionaries to the islands of the South Pacific that the desirability of religious pioneers possessing some knowledge of medicine and surgery first became apparent. There were, as we have said, no doctors there, nor had the people any means of alleviating their sufferings, whatever befell them ; and soon after the arrival of the emissaries of the London Missionary Society they were appealed to by one of the chiefs, who wanted to know if any of the new teachers could assist women in difficult labours. Unfortunately, none of them knew much of surgery or medicine, and they were obliged to admit that they could do no good. This was very unlucky. They professed to have power—what sort of power was it? If they had no power for good, it must be for evil. Shortly after their arrival three of the chiefs died, and the belief soon got abroad that these successive calamities were due to the prayers of the missionaries. Chiefs had never died so fast till they came, and if they continued their praying, very soon there would not be a chief left in the islands. Partly from this untoward experience, and partly from compassion for the sufferings of poor mortals exposed to all the ills that flesh is heir to, with none of the alleviations that modern science and philanthropy have provided, missionaries began from that time to turn attention to doctoring. Thus, in the darkest shades of heathendom as in the cold brilliance of the light of civilisation in Ancient Greece, medicine and religion have alike been ascribed to "the gods."

But apart from the teaching of Christian missionaries, medicine and religion have usually been associated in uncivilised lands. Very commonly the medicine-man and the priest and the juggler have been one and the same individual, and the ceremony of initiating a member of the fraternity is in many parts of the world a terrible ordeal, involving revolting cruelties to the unhappy novice himself. Many savages have a theory—truth to confess, some of us who call ourselves civilised secretly hold something like it —that a man who undertakes to cure pain ought properly to learn as much as possible about it by first enduring it himself. Very natural indeed. Who would not rather deliver himself over into the hands of a dentist who had had a tooth or two out himself, than to him who had never known what tooth-extraction really means? But the savage wants a doctor who not only knows what pain is, but can endure it unflinchingly. Some of the North American Indians initiate their medicine-men by a procedure which well-nigh kills them. The selected youth presents himself to a doctor of established reputation, who cuts four gashes about three inches long in his shoulders, and underneath the slits he has cut he passes a stick of smooth, hard wood, and then a buffalo thong, which he ties tightly. He serves the breast in the same way. One thong is now tied to a long pole and the other to some heavy weight, such as a buffalo skull. This being arranged, the candidate for medical status jumps into a vigorous dance, which he accompanies with an appropriate song. The consummation he has to aim at is the tearing of himself away from the heavy weight or the fixed horizontal pole by sudden leaps and plunges, and the more desperately he dances the sooner this is likely to be achieved. "At times," writes an eye-witness of this terrible ordeal, "the flesh on back and breast seems to stretch eight or ten inches, and, when pulled up, closes down again with a pop. This dancing continues until the flesh-fastenings break. The novitiate is by that time a terrible-looking object, and so nearly exhausted that he has to be helped away. His wounds are washed and bound up, presents are made to him, and he is thenceforth recognised as a medicine-man." On the whole, one cannot help thinking that our own medicine-men, who are said, some of them, to pay a thousand pounds in money and four or five years of their lives for a doctor's diploma, might be worse off, though it is true they might find among the Indians of North America a quicker entrance into the profession. Murray, a traveller among them, says that in some tribes nothing is easier than to become a medicine-man. "Any ignorant idler among the Pawnees, who takes it into his head to become a doctor, gives notice of it by assuming a solemn deportment, wearing his robe with the hair outwards, and learning to make a noise in the throat, which is distinctive of his profession, and which resembles the sound made by a person who is gargling for a relaxed uvula." Here it would seem his medical studies and accomplishments end, and his qualifications are judged of by the success attending his practice. This at least seems fair and reasonable, only one might be inclined to doubt the probability of his finding any patients, if it were not that here among ourselves there are plenty of medicine-men who do find patients enough to grow fat upon, and that without any better qualifications than a Pawnee Indian can show. Among various people there are many different ceremonies of initiation for medicine-men, and to some of these we shall refer in another paper.

*(To be continued.)*

